# The complete mitochondrial genome of *Helice Sheni* and its phylogenetic implication

**DOI:** 10.1080/23802359.2018.1524721

**Published:** 2018-10-26

**Authors:** YiMing Yuan, YanLong He, ShouHai Liu, Xiao Ji, YuTao Qin, XiaoBo Wang

**Affiliations:** a East China Sea Environmental Monitoring Center of State Oceanic Administration, Shanghai, People’s Republic of China;; b Key Laboratory of Integrated Monitoring and Applied Technology for Marine Harmful Algal Blooms, SOA, Shanghai, People’s Republic of China

**Keywords:** Mitochondrial genome, *Helice sheni*, phylogenetic relationship, comparative mitogenomic analysis

## Abstract

In this study, the complete mitochondrial genome (mitogenome) of *Helice sheni* was amplified and analyzed. The mitogenome is 16,062 bp in length, encoding the standard set of 13 protein-coding genes, 22 tRNA genes, 2 rRNA genes, and one control region. The nucleotide frequency of the mitogenome is as follows: A, 34.81%; C, 23.24%; G, 12.68%; and T, 29.25%. Eight overlapping areas and 22 intergenic spacers were found in the complete mitogenome. The typical initiation codon (ATT) and stop codon (TAA) were observed in eight genes, respectively. The phylogenetic tree indicates that *Helicana wuana* is closely related to *H. sheni*.

*Helice sheni* is widely distributed in China. However, limited research has been done on it. Mitochondrial DNA information is very important for species identification and phylogenetic analysis (Nishimoto et al. 2017). In this study, the complete mitochondrial genome (mitogenome) of *H. sheni* was firstly determined. Our results provide essential data for the studies of population genetics and phylogenetic analysis.

In this study, the samples of *H. sheni* (accession number B0041-1) were collected from Chongming, China. (122°0′13.68″, 31°28′18.53″). The specimen was stored in Sample Room of East China Sea Environmental Monitoring Center. Genomic DNA was extracted from muscle. The mitogenome was sequenced using Shot Gun Sequencing (Batzoglou et al. 1999). Then all the sequences obtained were analyzed and assembled using the software BioEdit (Hall 1999). The location of tRNAs was determined by using tRNA scan-SE 1.21 (Schattner et al. 2005).

The complete mitogenome of *H. sheni* (Genbank accession MH593562) was determined in this study. The length of the mitogenome is 16,062 bp, consisting of 13 protein-coding genes (PCGs), 22 tRNA genes, 2 rRNA genes, and one noncoding control region (D-loop regions). The overall base composition is 34.81%, 23.24%, 12.68%, and 29.25% for A, C, G, and T, respectively, with slight A–T bias (64.06%). The genes *ND1*, *ND4*, *ND4L*, *ND5*, *12S rRNA*, *16S rRNA*, *tRNA-Cys*, *tRNA-Gln*, *tRNA-His*, *tRNA-Leu*, *tRNA-Phe*, *tRNA-Pro*, *tRNA-Tyr*, and *tRNA-Val* are encoded on the light strand of the mitogenome, whereas the remaining genes are encoded on the heavy strand. In the complete mitogenome, 8 overlapping areas (22 bp in total) are observed and the length of overlapped sequence is 1–7 bp. A total of 22 intergenic spacers (536 bp in total) are found ranging from 1 to 93 bp. Seven pairs of genes are directly adjacent without intergenic or overlapping nucleotides. In general, ATG is the typical initiation codon. However, three PCG (*ATP6*, *ND3* and *ND6*) genes share the ATT initiation codon, while *ND1* and *ND2* have the ATA and ATC initiation codons, respectively. Most of the PCGs use TAA or TAG as the stop codon, while *COX1* uses an incomplete stop codon T. The 12S rRNA and 16S rRNA, with lengths of 813 and 1323 bp, respectively, are both located between *tRNA-Leu* and *tRNA-His*. And the length of 22 tRNA genes ranges from 62 to 76 bp.

To analyze the phylogenetic relationship among 15 species, a phylogenetic tree was constructed using Neighbor-Joining method by the software MEGA7 (Figure 1) (Kumar et al. 2016). The phylogenetic tree deriving from amino acid sequences of 13 PCGs. *Helice sheni* is closest to *Helicana wuana*, with *H. latimera* and *H. tientsinensis* being the sister taxon. In addition, *Eriocheir* is the closest taxa with *Helice* species. This study would provide basic data for genetic analyses in the future.

**Figure 1.**
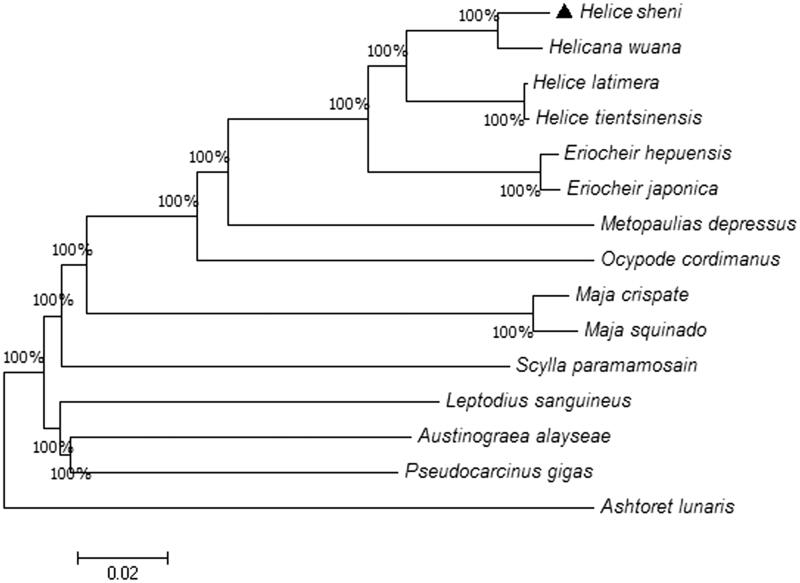
Neighbor-Joining tree of the amino acid sequences of 13 protein-coding genes. The numbers on the nodes show the bootstrap percentages. The genome sequence in this study is labeled with triangle.
